# Proposal for Handling of Medicine Shortages Based on a Comparison of Retrospective Risk Analysis

**DOI:** 10.3390/ijerph19074102

**Published:** 2022-03-30

**Authors:** Bela Turbucz, Martin Major, Romana Zelko, Balazs Hanko

**Affiliations:** 1 University Pharmacy Department of Pharmacy Administration, Semmelweis University, 1085 Budapest, Hungary; hanko.balazs@pharma.semmelweis-univ.hu; 2 Department of Oro-Maxillofacial Surgery and Stomatology, Faculty of Dentistry, Semmelweis University, 1085 Budapest, Hungary; major.martin@dent.semmelweis-univ.hu

**Keywords:** drug shortage, critical medicines, risk assessment, comparison, countermeasures

## Abstract

Introduction: We reviewed and compared current drug shortages and shortage management practices in six selected countries (Hungary, Belgium, Spain, Switzerland, Australia, United States) based on the most comprehensive national shortage databases for each country, for four Anatomical Therapeutic Chemical (ATC) groups, to analyze the criticality of drug shortages across countries and identify best practices in shortage management strategies. Materials and Methods: Countries were selected to cover a wide geographical range of high-income nations where a lack of economic power as a potential source of drug shortages is not observable. ATC groups were selected based on a pre-examination of the databases to analyze groups most often in shortage, and groups where the absence of which could have a severe negative impact on treatment outcomes. The bias originating from the different reporting systems had to be reduced to gain comprehensive and comparable information. The first bias-reducing mechanism was transforming the raw number of shortages into proportion per million people. Secondly, critical cases were classified, and thirdly, critical cases were compared with the Word Health Organization (WHO) Essential Medicine Lists. Results: The results indicate that every European country studied reports significantly higher total and critical shortages per population compared to the US and Australia. Within Europe, Hungary reports the highest number of cases both for total and critical shortages, while Spain has the lowest results in both aspects. While in the US and Australia critical shortages were observable in similar proportions across all ATC groups, in European countries ATC groups of anti-infectives for systemic use (J) and the nervous system (N) were found to account for a notably higher proportion of critical shortages. Current shortage management practices were examined in each country and classified into five groups to identify common best practices. Conclusions: Due to the different characterization of reporting systems, several bias-reducing mechanisms should be applied to compare and evaluate shortages. In addition, European harmonization should be initiated to create mutually acknowledged definitions and reporting systems, which could be the basis of good drug shortage handling practices in Europe.

## 1. Introduction

Drug shortages can be defined as when the available or calculated demand for drugs does not sufficiently meet the end-user level demand. The issue affects all stakeholders along the pharmaceutical supply chain, ranging from Marketing Authorization Holders (MAHs) through wholesalers to hospitals, community pharmacies, and patients, posing a serious threat to treatment outcomes [1]. Drug shortages are recognized as a global problem, to which local responses are necessary, taking into account regionally diverse factors [2]. However, international coordination and common best practices would be beneficial for more effective shortage management. As a starting point, internationally adopted guidelines and definitions would be useful to compare different regions, as currently these are lacking [3].

Competent authorities (CAs) have the most crucial role in handling shortages: they must ensure the continued volume of pharmaceutical products, and therefore they should always be aware of any interruptions in the supply of products and take actions to mitigate the impact of drug shortages [4]. Competent authorities worldwide took various actions over the past decades to collect, monitor, evaluate, and prevent drug shortages and reduce their impact on the healthcare system [5]. In Europe, the responsibility to avoid shortages is shared between authorities, MAHs, and distributors. According to Article 81 of Directive 2001/83, MAHs and the distributors shall ensure appropriate and continued supplies of the medicinal products to pharmacies and hospitals to address the needs of patients [6]. The European Association of Hospital Pharmacists (EAHP) started investigating drug shortages in 2012 due to increased reports from its members expressing difficulties in sourcing medicine [7]. In the United States (US), President Obama signed the Food and Drug Administration (FDA) Safety Innovation Act in July 2012 [8]. The Act mandated manufacturers to inform the FDA of impending shortages to enable early mitigation of the problem. In Australia, the Therapeutic Goods Administration (TGA) dedicated a website to monitoring medicine shortages in May 2014 [9]. However, drug shortages remain a global issue, also present in most developed countries, and competent authorities should further improve their shortage management practices, as the substitution or absence of safe and effective therapies can compromise medical procedures, cause errors, and have a serious adverse impact on patient care. This is especially important in the case of drugs included on the World Health Organization (WHO) essential medicine list, as their shortage is more likely to have severe negative impacts on treatment outcomes [10].

Drug shortages arise due to several root causes, which can be grouped into economic factors, manufacturing and quality factors, and logistic and regulatory factors [2]. Therefore, a diverse set of actions is necessary to effectively address them [11].

This study performs a comparative analysis of six selected countries to understand how total reported shortages and critical shortages compare internationally, and which ATC groups are mostly affected by critical shortages. The study also reviews shortage management practices already in place to reduce shortages and mitigate their impact, with the aim of identifying best practices.

## 2. Materials and Methods

To carry out the analysis, countries and respective databases have been selected according to pre-defined criteria. As a next step, databases have been processed focusing on selected therapeutic categories, the shortage of which would have the most severe impact on patient care. Finally, risk assessment has been carried out to identify critical shortages. In the analysis, both total and critical shortages have been compared across countries and ATC groups.

### 2.1. Criteria for Selecting Countries

Six countries have been selected for the analysis to cover a wide geographical range and allow for diversity. The selected countries are Australia from the Pacific region, the United States from North America, Belgium, Spain, and Hungary from the European Union (EU), and Switzerland, being a European country not part of the EU. As countries use diverse reporting systems and publish different information on shortages, the selection criteria has been developed to filter out countries where not enough details are public to carry out risk assessment and identify the ATC group for each shortage.

The following aspects were taken into account throughout the country selection method:

- First world countries, where drug shortages are not a result of the lack of available financial resources.
- Have a profound pharmaceutical industrial background.
- Publicly available reporting system.
- Reported shortages must be classifiable according to the Anatomical Therapeutic Chemical (ATC) classification system.
- Information is public regarding available substitutes to allow for assessment of severity.
- Discontinued presentations listed separately from current shortages.

The six selected countries satisfy all the above-mentioned criteria and therefore constitute a profound ground for analysis.

### 2.2. Processing the Databases

To process analogous and comparable information on shortages in the chosen countries, shortage databases of all countries were accessed in the one-week time period between 4 and 9 March 2021. As most databases do not offer the possibility to access detailed data on past shortages but only show current shortages, longitudinal analysis was not possible. In Hungary, Belgium, Spain, and Australia, one national database was available, managed by the competent authorities. In two countries, the United States and Switzerland, two or more databases were available, run by multiple authorities (US) or also by private bodies (Switzerland). In the US, The American Society of Health-System Pharmacists (ASHP) database has been chosen [12], as it presented more details regarding the available substitutes compared to the FDA reporting system. In Switzerland, the Martinelli database was chosen as the national competent authority’s database focuses only on a narrow scale of medicines considered essential by law [13].

Shortages reported because of discontinuation of production, stopped commercialization, or the interruption of commercialization were excluded from the analysis. This was necessary because there was not enough information regarding the reason and the duration of these shortages.

### 2.3. Therapeutic Categories

Shortages were classified according to the ATC Classification System rules, which classify the active ingredients of drugs according to the organ or system on which they act and their therapeutic, pharmacological, and chemical properties. The World Health Organization Collaborating Centre (WHO CC) controls it for Drug Statistics Methodology [14]. We have chosen the following particular ATC groups in shortage for further examination.

- C: Cardiovascular system
- L: Antineoplastic and immunomodulating agents
- J: Anti-infectives for systemic use
- N: Nervous system

These four ATC groups have been selected for analysis, as these categories of medications play a significant role in the therapeutic arsenal. The permanent or temporary absence of these medicines would cause a serious impact on patient care and the healthcare system [15,16,17,18]. The selection of these groups has been confirmed with the pre-examination of the data, which showed these ATC groups present are in the highest proportions among all reported shortages.

### 2.4. Allocation of Risk Assessment in Studied Countries

We have investigated all the posted products “currently in shortage” one-by-one to determine whether the risk is considered acceptable or critical. The different presentations of a particular pharmaceutical ingredient were counted as individual shortages. The aim was to assess the severity and separate critical and non-critical shortages. This is key, as the ultimate purpose of every reporting mechanism and countermeasure is to reduce critical shortages. Table 1 summarizes how critical shortages were identified for each country. In most of the studied countries, authorities did not publish a severity assessment, and therefore, criteria to distinguish critical cases had to be defined. The criterium was if no domestic alternatives were available, the shortage was considered critical, as in this case emergency imports would be necessary. It is general practice in every country that the competent authority suggests a domestic alternative for every drug that is in shortage. If no domestic alternatives are available, the country can perform an emergency import from abroad, but this is considered a high-risk and high-expense solution, which is to be avoided. In Belgium and Australia, the database already included some information regarding the severity, which has been augmented to match the criteria applied for all other countries.

### 2.5. Comparison of the Extent of Different Shortages

To avoid significant populational bias, instead of comparing nominal shortage numbers, the proportion of critical shortages to the country population was taken into consideration. The ratio was calculated as the number of shortages per million people [23].

### 2.6. Binomial Probability Tests of Proportion of Critical Shortages across ATC Groups

Data have been analyzed to obtain insights into critical shortages and understand whether the proportion of critical shortages differs across the four ATC groups studied (C, J, L, N). We have used the software “Stata” version 17.0 for the analysis (StataCorp LLC 4905 Lakeway Drive, College Station, TX, USA).

For the purpose of statistical analysis, the variable Critical_BI was defined, which is an indicator variable taking the value 1 for shortages that are critical, and 0 for the shortages that are non-critical. Our null hypothesis was that the proportion of critical shortages will not be significantly different across ATC groups.

## 3. Results

**Figure 1:**
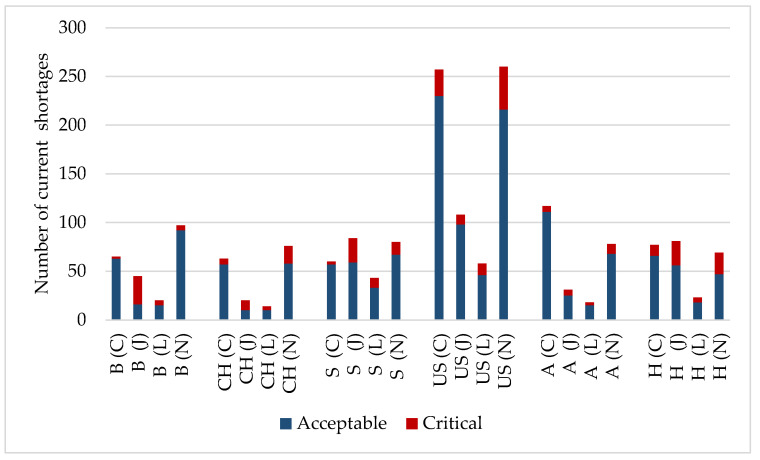
Number of shortages according to ATC groups (including both critical and non-critical). B: Belgium; CH: Switzerland; S: Spain; US: United States; A: Australia; H: Hungary. ATC groups are shown in brackets (C, J, L, N).

The number of shortages derived from the databases regarding the above-mentioned four ATC groups have been analyzed both quantitatively and qualitatively. Our aim regarding the quantitative analysis was to compare total and critical shortages per million people. The qualitative evaluation aimed to compare the proportion of critical shortages by ATC groups per million people. The number of total shortages for the four analyzed ATC groups were obtained for the six chosen countries. Figure 1 shows the shortages in the studied countries, including critical ones. Nevertheless, as Table 2 outlines, the characteristics of reporting systems differ significantly across countries, as they are built on different conceptions regarding the reporting obligation. To obtain comparable data and conduct a reliable analysis, three bias-reducing steps have been performed.

**Figure 2:**
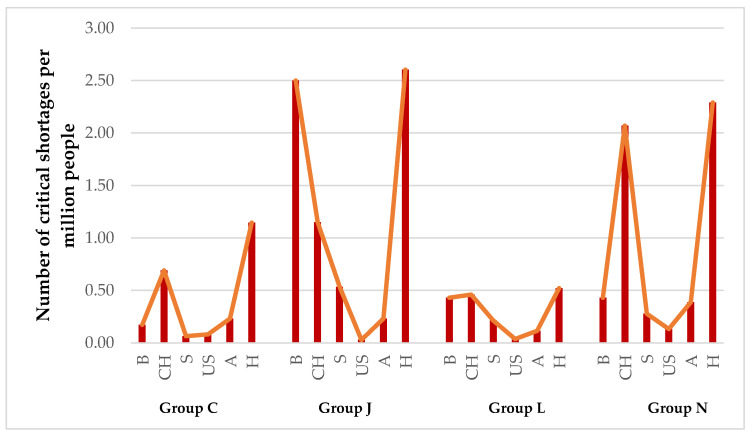
Comparison of critical shortages of various countries by ATC groups.

The bias-reducing steps were the following:

1. Transforming the data into population-proportionate figures.
2. Filtering out critical cases from all shortages (according to criteria in Table 1).
3. Comparing critical cases with the WHO Essential Medicine List.

These steps were all necessary so that despite the differing reporting systems, some comparative international oversight could still cover drug shortages. The lack of uniform definitions, reporting systems, and severity assessments along unified international criteria creates significant obstacles to any international comparison of shortages. The characteristics of the national reporting systems of the countries under analysis are highlighted in Table 2.

To perform the quantitative analysis of the data, the first two bias-reducing steps were carried out. Data have been transformed into population-proportionate figures for all countries, and critical cases have been filtered out from all shortages according to the predefined criteria described in the Materials and Methods Section of this paper. As a result, Table 3 was developed. Column (d) of Table 3 shows the calculated proportion of total shortages per million people for each country. The highest proportion was recorded in Hungary and the lowest in the US. The average of the European countries for total shortages per million people (d) is significantly higher than non-European figures. The Spanish figure is an outlier compared to other European countries, as shortages per million people (d) are at least 75% less than any other European country. However, comparing shortages per million people across countries still does not afford unbiased results, as the total number of shortages reported can largely differ across countries due to the reporting system in use. To obtain less biased and more relevant results, the proportion of critical shortages per million people has been calculated in column (e). These figures still reflect that the proportion of critical shortages per population is higher in every European country than the non-European figures. The percentage of critical shortages among all reported cases is shown in column (f). The percentage of critical shortages is on average two times higher in Europe than in the US or Australia. This observation is crucial, as the ultimate purpose of every reporting mechanism and countermeasure is to reduce the number of critical shortages in a given country.

Table 4 shows that the proportion of critical shortages is 16.87% across the whole sample. In contrast to our hypothesis, the binomial probability tests performed for each ATC group (ATC group is denoted by *ATC_ID*) indicated significant differences in the shortage proportions of certain ATC groups. Table 4 displays the result that the observed proportion (*Observed p*) is significantly different from the expected proportion (*Expected p*) for 3 out of 4 groups. In therapeutic group C, only 8.6% of total shortages are critical, while group J seems to be the most affected by critical cases (28.5% of observed shortages are critical). The proportion of critical shortages observed is 22.2% for group L and 17% for N, only slightly higher than the average across the sample.

Knowing that the proportion of critical shortages is significantly different across ATC groups, the next aim was to understand whether and how these differences vary across countries, taking into account the differences in population. Figure 2 outlines the proportion of critical shortages across the countries in specific ATC groups. A pattern can be observed regarding the relative distribution of shortages across the investigated ATC groups, except for a few outstanding data points. It is in line with our expectations based on the total number of critical shortages in Table 3 that the proportion of critical shortages per million people was significantly higher in Switzerland and Hungary in each ATC group, even compared to the European average. Similarly, the lowest proportion of critical cases for each ATC group was observed in the US. A pattern that can be observed is that the distribution of critical shortages across ATC groups for the US and Australia is relatively steady, and no ATC group shows significantly higher proportions of critical shortages. The opposite is true for Belgium, Hungary, and Switzerland, where the proportion of critical shortages is outstandingly higher in therapeutic group’s J and N, anti-infectives for systemic use and nervous system drugs. It is apparent that in European countries with high total numbers of shortages per population, these two groups are causing critical pharmaceutical gaps. Our results are in agreement with the surveys from the European Association of Hospital Pharmacists [7] and the American Society of Health-System Pharmacists [32], who state that in the group of anti-infectives, shortages are particularly an outstanding issue due to the high ratio of medication errors, the increasing antimicrobial resistance, the substandard patient outcome, and the lack of development of new antibiotics [33].

As the final step of the analysis, critical shortages were compared to the WHO Essential Medicine List. The results are presented in Table 5. The World Health Organization proposed the concept of essential medicines in 1977, which is a catalogue of every healthcare system’s minimum medicine needs. Essential medicines are those that satisfy the priority healthcare needs of the population. The basic concept is that high-priority drugs should be available as part of a functioning health system for all people, guiding physicians to evidence-based and rational prescribing [34]. Clinical evidence confirms that medicines included in the list can significantly improve patients’ outcomes and their shortage can have a severe negative impact on treatment quality [35]. Therefore, it should be a key priority to keep the shortage levels of medicines deemed essential by the WHO as low as possible.

Table 5 demonstrates what percentage of all critical shortages are included on the WHO Essential Medicine List by country. The goal of every healthcare system should be to keep this ratio as low as possible, as a WHO essential drug being in critical shortage would mean that the country has no domestic alternative to replace the medication and an emergency import is necessary. Table 5 shows that Switzerland and the US are performing best in this aspect, with 36.8% and 45% of critical shortages being WHO essential medicines, respectively. Belgium displays the worst result, with over 90% of its critical shortages being WHO essential. The results show no correlation between the volume of critical shortages per population and the percentage of WHO essential medicines among critical shortages. Countermeasures should be implemented to reduce both the number of critical shortages and specifically, the WHO essential drugs in critical shortage.

## 4. Discussion

### 4.1. Comparison of Shortage Management in Examined Countries

The analysis clearly shows that the proportion of critical shortages relative to population size is significantly higher in Europe than in other investigated regions of the world. This affects every ATC group under examination, but anti-infective (J) and nervous system (N) drugs show drastically higher critical shortage levels in Europe. Our findings are in agreement with the study from 2018 conducted by Videau in hospitals, where Switzerland was one of the most and Spain the least affected country by shortages in both studies, and anti-infective medications were the most affected therapeutic group [5]. The critical shortages of medicines on the WHO Essential Medicine List need to be reduced and prevented in particular. European nations need to adopt a wider range of transparent, unified shortage management countermeasures across countries to address drug shortages. This section of the paper reviews the various forms of countermeasures already in place in some of the examined countries. A selection of these could be unified and extended over the whole of Europe. The current forms of countermeasures by country are reported in Table 6 and can be grouped under the six following categories:

1. Compulsory stockpiling
2. Measures for essential medicines
3. Notification responsibility
4. Measures affecting wholesalers
5. Export bans
6. Emergency imports

### 4.2. Compulsory Stockpiling

In Australia, the National Medical Stockpile must maintain “key medicines” to avoid critical shortages of essential drugs [30]. A similar system exists in Switzerland, where the government defines medications that are subject to compulsory stockpiling in the appendix to Ordinance and the level of such stock that would satisfy average domestic consumption for three months without any import [40].

### 4.3. Measures for Essential Medicines

In Spain, the government can demand production and commercialization from MAHs to bridge supply gaps concerning essential products [52]. This seems to be an effective measure to cut back shortages, as critical shortages per million people in Spain were significantly the lowest among European countries. The US shortage management system works similarly. In response to the shortage of a medically necessary drug, the FDA can suggest and support establishing a new manufacturing site or even involving a new supplier. If a MAH decides on a voluntary recall, the FDA may conduct risk evaluation and encourage other MAHs to initiate, maintain, or increase production of the drug [46]. They can also facilitate the review of new generic applications that are potential alternatives [46]. The regulation also permits hospitals within the same health facility to repackage drugs into smaller units to alleviate drug shortages [8]. In Spain, MAHs that stop the distribution of a medicinal product without the authorities’ permission face heavy fines [53].

### 4.4. Notification Responsibility

In the US, authorities maintain an apparent oversight of shortages. They, therefore, can handle them very effectively—the quantitative analysis clearly reflected that the critical shortages per million people are the lowest in the US. The criteria for products reported in the central systems are strict and well-defined, so authorities can monitor developing shortages from an early stage and address them accordingly [12]. For example, MAHs are mandated to inform the FDA of impending shortages six months in advance when they plan to stop producing a single-source or medically necessary (life-supporting, life-sustaining, or intended for use in the prevention or treatment of a debilitating disease or condition, including emergency medical care or during surgery) drug [8]. This effectively helps to maintain a low percentage of shortages that turn into critical. The FDA may also oblige the MAHs to conduct periodic risk assessments to address vulnerabilities in its supply chain.

### 4.5. Measures Affecting Wholesalers

In Belgium and Spain, some countermeasures specifically target wholesalers, who are obliged to ensure continued and adequate supply. In Spain, all wholesalers are required to deliver within 24 working hours [38]. In Belgium, to reduce the cases where shortages arise due to “distribution problems”, distributors were assigned as full-line and regular wholesalers. MAHs are obliged to supply within a shorter period to full-line wholesalers. Full-line distributors are required to deliver emergency shipments in 24 h. They must have a range of specified medicines in stock to supply the needs of defined geographic areas. Moreover, full-line distributors are only allowed to supply strictly determined wholesalers, domestic pharmacies, and hospitals; thereby, parallel export is not permitted [36].

### 4.6. Export Bans

In Australia, export can only be performed by MAHs or designated distributors acting on behalf of the MAHs, not by most wholesalers like in many European countries [49]. This is highly important, as parallel export is usually a key factor in causing shortages, mostly affecting countries with low drug prices compared to international averages. In Spain, authorities (AEMPS) can restrict exportation only to medicinal products without therapeutic equivalents [26]. There are serious penalties and fines of up to 1 million euros for distributors who export medicines when this activity has been forbidden.

### 4.7. Emergency Imports

When there is no other possibility to resolve a critical shortage as no substitution is available domestically, emergency imports must be performed. The conditions of an emergency import are determined by national laws. In most countries, this is contingent on the approval of the competent authority, such as the FDA in the US, the OGYEI in Hungary, or the Spanish Agency of Medicinal Products and Medical Devices in Spain (see in Table 6). In Belgium, wholesalers may also perform emergency imports from the EU based on a doctor’s request, in the specific quantities necessary for the given treatment [39].

## 5. Limitations

The findings are limited by the high degree of incomparability of data on drug shortages across countries. As reporting criteria and reporting systems used significantly differ in every country examined, it has been hard to develop a comprehensive and comparable overview of shortages in each country (including the number, severity, causing factor, and possible solution of all shortages). The authors are aware that the analysis does not contain deep statistical research to support insights. The cause of this is that only publicly available databases could be recruited, which contain only limited and categorical data. Even the data fragments provided highly differ across countries. Publicly available, internationally uniformed, and frequently updated databases containing numerical data (duration of shortage, cost of alternative therapy, number of patients using medication, effectiveness of particular countermeasures) would be necessary to perform a more accurate statistical analysis on the topic. The criteria which define shortages to be reported towards authorities can make large differences in the data, e.g., in Spain, shortages where “quick solution is available” are not noted in the reporting system, which can significantly alter the results of the cross-country comparison. In Australia, the lower proportion of critical shortages could be explained with the definition complexity as it considers both the number of alternatives and the potential impacts on patients. In Switzerland and the US, multiple databases of shortages are available offering different details, maintained by various authorities (FDA and ASHP) or organizations (Federal Office of Public Health, Swissmedic, Federal Office for National Economic Supply, Martinelli Consulting). This is an issue because large discrepancies among the various databases and varying definitions limit transparent international comparison.

## 6. Conclusions

The following recommendations should be considered to handle the issue of drug shortages more effectively in Europe. Since drug shortages do not respect borders and cross-country collaboration would be beneficial for more effective shortage management, a unified European definition and reporting criteria of shortages would be necessary to assure internationally consistent monitoring, reporting, comparisons, responses, and solutions. National authorities across Europe should be aware of shortages through coordinated systems, increasing cross-country transparency, and facilitating solutions in every country. It was a significant step forward in the European Union that in July 2019, the EMA published the “Guidance on detection and notification of shortages of medicinal products for Marketing Authorisation Holders in the Union” [54]. The document contains the effort to facilitate the more uniform reporting and communication of drug shortages and create a harmonization “drug shortage” definition. The document “Good practice guidance for communication to the public on medicines’ availability issues” [55] contains communication guidelines for the national authorities and the EMA for patients and healthcare professionals.

The ideal unified definition for critical shortages should positively discriminate products included on the WHO Essential Medicine List, should take into account the number of alternatives, and should determine the exact start and end dates of a shortage. International harmonization should be initiated to create mutually acknowledged definitions and reporting systems, which could be the basis of good drug shortage handling practices in Europe.

## Figures and Tables

**Table 1:** Allocation of critical shortages.

Country	Allocation of Critical Shortages
Switzerland	If there is no domestic alternative [13].
Spain	If there is no domestic alternative [19].
Hungary	If there is no domestic alternative [20].
US	The “shortage risk index” was defined with the ratio of unavailable and available presentations. If this number is higher than 5, the shortage is considered critical. If the database states: “there are no presentations available” or “there is insufficient supply for usual ordering”, despite that the index will be lower than 5, the shortage had been considered critical.
Belgium	According to the FAMHP decision tree and in case it is necessary to import from abroad [21].
Australia	According to the TGA definition, if the medicine is included on the Medicines Watch List (MWL) or if the shortage has the potential impact to have a life-threatening or serious impact on patients and there is not likely to be a sufficient supply of potential substitutes automatically considered as critical [22].

**Table 2:** Characteristics of notification systems.

Country	Manager of Database	Definition of Shortage	Source of Information	Scope of Report	Countermeasures
**Belgium**	Federal Agency for Medicines and Health Products (FAMHP)	Unable to deliver for an uninterrupted period of four days [24].	MAH should notify the FAMHP within 7 days after the start of the unavailability when a drug will be unavailable for longer than 14 days [21].	Concerns about a certain presentation. The entire range of medicine is not unavailable.	Emergency import Greater responsibility to full-line distributors MAH should give an exact reason and compensate costs
**Switzerland**	Martinelli Consulting GmbH	Supplies not satisfying demand and orders [13].	The website is based on voluntary reports from companies and users [13].	Drugs officially approved in Switzerland are listed in Martinelli database.	Emergency import Strategic stockpiling Define essentiality
**Spain**	Spanish Agency of Medicines and Medical Products (AEMPS)	The number of available units is below the level of national or local consumption needs [25].	AEMPS database lists current or anticipated supply problems for different presentations. If a quick solution is expected, not included in the list [19].	Concerns about a certain presentation. The entire range of medicine is not unavailable [26].	Define essentiality Emergency import Maintain MA and production Delivery in 24 working hours Export ban
**US**	American Society of Health-System Pharmacists (ASHP)	Supply issue that affects how a pharmacy prepares, dispenses a drug, or influences patient care when prescribers must use an alternative agent [10].	Voluntary reports from practitioners, patients, and others [12].	ASHP lists every drug shortage reported through the online report form as soon as it is investigated and confirmed, usually within 24–72 h [12].	Define essentiality Emergency import Reevaluates voluntary recalls Expedite changes Maintain MA and production Drugs into smaller units ASHP management practice
**Australia**	Therapeutic Goods Administration (TGA)	The supply of medicine will not (or may not) meet the demand for the medicine at the subsequent six months, including all patients who take (may need to take) [27].	MAHs are required to report all registered medicines in 2–10 days upon severity [22].	The medicines are set out in Therapeutic Goods Determination [28].	Emergency import Export registration National Medical Stockpile [29] Medicine Watch List (MWL) [30].
**Hungary**	National Institute of Pharmacy and Nutrition (OGYEI)	If the MAH is unable to maintain adequate and continuous supplies of specific medicinal products, or unwilling to supply the preparation temporarily or permanently [31].	Before the final delivery to the wholesaler, but in a maximum of two months [31].	Concerns about a certain presentation. The entire range of medicine is not unavailable.	Recommending alternatives Shortage declaration by CA Emergency import Export ban

**Table 3:** Number of shortages per million people in the studied countries, including critical cases.

Country	Date	No. of Shortages (a)	Considered as Critical (b)	Population in Millions (c)	Shortage per Million People (d = a/c)	Critical Shortage per Million People (e = b/c)	Percentage of Critical/All Shortages (f = e/d)
Belgium	4 Mar 2021	227	41	11.6	19.57	3.53	18.1
Spain	6 Mar 2021	267	51	46.7	5.72	1.09	19.1
Hungary	9 Mar 2021	250	63	9.6	26.04	6.56	25.2
Switzerland	6 Mar 2021	173	38	8.7	19.89	4.37	22.0
Average of examined European countries	-	229.25	48.25	-	17.805 *	3.89	21.1
US	9 Mar 2021	683	93	332.9	2.05	0.28	13.6
Australia	8 Mar 2021	244	25	25.8	9.46	0.97	10.2

* The European average values were calculated from the data of individual European countries examined.

**Table 4:** Proportion estimation of critical shortages over the whole dataset. Binomial probability tests of proportion of critical shortages across ATC groups.

Critical_BI	Proportion	Std. Err.	Logit (95% Conf. Interval)		
0	0.8313449	0.0087199	0.8135458	0.8477629	
1	0.1686551	0.0087199	0.1522371	0.1864542	
**ATC**	**Number of All Shortages (N)**	**Observed (k)**	**Expected (k)**	**Expected (p)**	**Observed (p)**
C	639	55	107.7706089	0.16866	0.08607
J	369	105	62.2337319	0.16866	0.28455
L	176	39	29.6832976	0.16866	0.22159
N	660	112	111.312366	0.16866	0.16970

**Table 5:** Summary of critical shortages of the examined countries according to the WHO Essential Medicine List.

	Belgium	Switzerland	Spain	Hungary	Average of Examined European Countries	US	Australia
**No. of critical shortages/million people**	3.53	4.37	1.09	6.56	3.89	0.28	0.97
**No. of critical shortages on WHO Essential List/million people**	3.19	1.61	0.75	3.96	2.38	0.28	0.74
**WHO Essential/all critical shortages (%)**	90.36	36.82	68.76	60.34	64.07	45.06	75.92

**Table 6:** Countermeasures introduced by countries. * Not applicable means that we did not obtain relevant literature on this topic.

	Compulsory Stockpiling	Measures for Essential Medicines—Legal Definitions	Notification Responsibilitiy—Legal Background	Measures Affecting Wholesalers	Export Bans	Emergency Imports
**BE**	Full-line wholesalers required to have a range of products in stock for the needs of the given geographic territory/ies [36].	Not defined.	Not providing the exact reasons is considered a clear legal violation [37].	Full-line wholesalers are assigned besides regular ones with special responsibilities and privileges [36,38].	Temporarily to medicinal products for which a shortage is notified [37].	Based on a doctor’s request wholesalers may temporarily import medicine from the EU, if no substitutes are available in BE, in specific quantities requested by the doctor [39].
**CH**	Delegated to companies at a defined level of stocks in Ordinance [40].	Compounds for which there are no or only limited substitutes have been affected by a supply shortage over the previous three years [40].	Determined in 531.215.32 Ordinance [40].	Managing the strategic level of inventory stock delegated by the federal government in local law [40].	Parallel export does not become significant due to high prices [41].	Upon application for temporary import submission [42].
**ES**	CA may demand commercialization to grant the suspension, or the revocation of the product [43].	Essential if the pharmaceutical gap cannot fully cover or have a high economic impact. Critical if it has no available therapeutic alternatives and has a complex manufacturing process, and/or has only one supplier [38].	Spanish Medicine and Products Devices Agency Circular No. 3/2011 [44].	All wholesalers are required to deliver within 24 working hours [38].	If lack of medicinal products causes a pharmaceutical gap [45].	The Spanish Agency of Medicinal Products and Medical Devices can also approve the import of medicines labeled in other languages or with an expiry date shorter than 6 months [38].
**US**	FDA supports MAHs to maintain production [46].	If used to treat or prevent a serious disease or condition, and there is no other adequate available source [47].	Safety and Innovation Act in 2012 [8].	* Not applicable.	* Not applicable.	FDA may allow emergency importation [46].
**A**	National Medical Stockpile maintains the strategic reserve of products [48].	Included on Medicines Watch List, has a potential life-threatening or serious impact, or has no potential substitutes [22].	Therapeutic Goods (Reportable Medicines) Determination 2018 [28].	Wholesalers also have a duty to notify authorities about the expected duration of a discontinuation [29].	Only for MAH or designated entity [49].	Therapeutic Goods Act has been amended to assist import [27].
**HU**	Products decreed by the minister should be available in the quantity defined therein [31].	Not defined.	Act XCV of 2005 on Medicinal Products for Human Use [31].	Authorized wholesale distributors shall be required to procure and supply the medicinal products as their authorization for wholesale distribution pertains [31].	The active substances decreed by the minister for a period not exceeding one year [31].	On the wholesaler’s request “contingent-approval” [50] or the physician statement “individual approval of OGYEI” [51].

## Data Availability

Not applicable.
